# *PMEL* p.Leu18del dilutes coat color of Kumamoto sub-breed of Japanese Brown cattle

**DOI:** 10.1186/s12864-022-08916-8

**Published:** 2022-10-07

**Authors:** Satoshi Kimura, Touko Hatakeyama, Takashi Koutaka, Kazuhiro Kubo, Satoru Morita, Keiko Eguchi, Kohji Saitoh, Kenji Yamauchi, Saki Imai, Atsushi Kashimura, Toshiaki Inenaga, Hirokazu Matsumoto

**Affiliations:** 1 Course of Agricultural Science, Graduate School of Agriculture, Tokai University, Kumamoto, Japan; 2 Department of Animal Science, School of Agriculture, Tokai University, Kumamoto, Japan; 3 Kumamoto Office, Phoenix College, Tokai University, Kumamoto, Japan; 4 Kumamoto Prefectural Agricultural University, Kumamoto, Japan; 5 Kumamoto Prefectural Agricultural Research Center, Kumamoto, Japan; 6 Kumamoto Station, National Livestock Breeding Center, Kumamoto, Japan

**Keywords:** Coat color dilution, DNA marker, Japanese Brown cattle, Kumamoto prefecture, *PMEL*, Whole genome analysis

## Abstract

**Background:**

Coat color is important for registration and maintenance of livestock. Standard coat color of Kumamoto sub-breed of Japanese Brown cattle is solid brown, but individuals with diluted coat color have been observed recently. In this study, we attempted to identify polymorphism(s) responsible for coat color dilution by whole genome analysis.

**Results:**

One of the diluted cattle possessed 7302 exonic polymorphisms which could affect genes’ function. Among them, 14 polymorphisms in 10 coat color-related genes were assumed to be specific for the diluted cattle. Subsequent genotyping with three diluted cattle and 74 standard cattle elucidated that *PMEL* p.Leu18del was the causative polymorphism for coat color dilution in this sub-breed. Individuals with del/del type of this polymorphism showed diluted coat color, but coat color of heterozygotes were intermediate with various dilution rates.

**Conclusions:**

Coat color dilution of Kumamoto sub-breed was caused by *PMEL* p.Leu18del. The causative del allele has been detected in several genetically distant cattle breeds, suggesting that *PMEL* p.Leu18del can be used as a DNA marker to control cattle coat color.

**Supplementary Information:**

The online version contains supplementary material available at 10.1186/s12864-022-08916-8.

## Background

Coat color is important for registration of livestock and this phenotype is artificially controlled. Japanese Brown cattle, one of Wagyu breeds, is divided into two sub-breeds, Kumamoto and Kochi sub-breeds. Although coat colors of both sub-breeds are brown as their names represent, their patterns are slightly different. Coat color of Kumamoto sub-breed is solid brown, while extremities of Kochi sub-breed are black [1, 2]. However, individuals with diluted, white coat color in Kumamoto sub-breed have been found recently. Abnormal coat colors prevent from registration; coat color dilution in Kumamoto sub-breed has a negative influence on their characteristics. Therefore, identifying the responsible polymorphism(s) for coat color dilution of this sub-breed is required.

Plural genes have been reported to produce white coat color. These genes are involved in various phenomena such as melanin synthesis, melanosome transport, and melanocyte development and differentiation [3]. For example, mutations in *TYR* (*Tyrosinase*) gene and its related genes can stop melanin synthesis, leading to diluted coat color called albinism in various species [4–7]. Genes associating with protein-actin complexes controlling melanocyte transport are other candidates for coat color dilution. Mutations in *Mlph* (*Melanophilin*), *Myo5a* (*Myosin VA*), and *Rab27a* (*RAB27A, Member RAS oncogene family*) show diluted phenotype in dogs, mice, and humans, respectively [8–10]. MITF (Microphthalmia-associated transcription factor) is a transcription factor that regulates coat color-related genes, which mutations have also been shown to cause coat color dilution in mice and horses [11, 12]. In cattle, two polymorphisms (p.Leu18del and p.Gly22Arg) in *PMEL* (*Pre-melanosome protein*) gene, associating with melanin deposition, have been identified as candidate polymorphisms responsible for coat color dilution (OMIA001545–9913). Because of the large number of gene types involved in this phenotype, whole-genome analysis was considered to identify candidate genes responsible for coat color dilution in Kumamoto sub-breed.

In this study, we conducted whole-genome analysis by next-generation sequencing in order to elucidate the responsible polymorphism(s) for coat color dilution in Kumamoto sub-breed of Japanese Brown cattle. This analysis was conducted under the hypothesis that the coat color dilution in Kumamoto sub-breed might be inherited recessively, due to relatively low frequency of diluted cattle. Subsequently, effects of candidate polymorphisms on coat color were analyzed by genotyping. The polymorphism identified in this study can be used as a DNA marker to control cattle coat color.

## Results

### Whole genome sequencing

The whole genome sequencing identified 21,409 polymorphisms in exonic regions of the diluted cattle. Among them, 7302 polymorphisms (6930 missense mutations, 326 frameshift mutations, and 46 nonsense mutations) were predicted to harm genes’ function. Sixty-six of them might affect coat color, according to “Color Genes” [13, 14] and “The Colors of Mice: A Model Genetic Network” [15].

The diluted cattle possessed 28 homozygous coat color-related polymorphisms, and, among them, 14 polymorphisms in 10 genes were assumed to be the diluted cattle-specific (Table S1). We regarded the polymorphisms in *USP13* (*Ubiquitin-Specific Protease 13*), *LYST* (*Lysosomal Trafficking Regulator*), and *PMEL* genes as candidates for coat color dilution (Table 1). *USP13* gene encodes a deubiquitinating enzyme that regulates MITF stability, one of the responsible factors for coat color dilution [11, 12, 18]. *LYST* gene is the responsible for Chediak-Higashi syndrome, an autosomal recessive bleeding disorder with coat color dilution in Japanese Black cattle, and Arg allele of p.His2015Arg leads its onset [27]. Mutations in *PMEL* gene have been reported to cause coat color dilution in various species [28]. In cattle, p.Leu18del and p.Gly22Arg, have been suggested to cause coat color dilution [29, 30]. Therefore, polymorphisms in these genes (*USP13* p.Met1Val, *PMEL* p.Leu18del, p.Gly22Arg, p.Ser36Leu, p.Ala612Glu, and *LYST* p.His2015Arg, p.Ala2575Val), were selected for subsequent analysis.

**Table 1:** Coat color-related genes with diluted cattle-specific DNA polymorphisms identified by whole-genome analysis

BTA	Gene	Phenotype of mutation mouse	References
1	*SOX2*	Yellow coat color	[16, 17]
	*USP13*	MITF abnormality?	[18]
3	*LMX1A*	White belt, white spot coat color	[19]
	*DOCK7*	white spot coat color	[20]
5	*KRT75*	Irregular aggregation of pigments	[21]
	*PMEL*	Diluted coat color	[22]
24	*SMCHD1*	Yellow coat color	[23]
28	*LYST*	Diluted coat color	[24]
X	*GPC3*	White spotting, belly spot coat color	[25]
	*NDP*	hyperpigmentation of the retinal pigment epithelium	[26]

### Identification of the responsible polymorphism for coat color dilution

To confirm specificity of the polymorphisms, genotyping with three diluted cattle and 74 standard cattle was performed. This genotyping excluded *USP13* and *LYST* genes from the candidates (Table 2). The genotypes of *USP13* p.Met1Val and *LYST* p.Ala2575Val were different among the diluted cattle. Although the diluted cattle had His/His type of *LYST* p.His2015Arg commonly, this genotype does not cause coat color dilution [27]. Additionally, two cattle with standard coat color were Arg/Arg type, suggesting that this polymorphism might not dilute coat color of Kumamoto sub-breed.

**Table 2:** Genotypes of candidate polymorphisms for coat color dilution in Kumamoto sub-breed of Japanese Brown cattle

Polymorphism	Genotype	Diluted (*n* = 3)	Standard (*n* = 74)
*USP13* p.Met1Val	Met/Met	–	39
	Met/Val	2	26
	Val/Val	1	9
*PMEL* p.Leu18del	Leu/Leu	–	65
	Leu/del	–	9
	del/del	3	–
p.Gly22Arg	Gly/Gly	3	39
	Gly/Arg	–	18
	Arg/Arg	–	17
p.Ser36Leu	Ser/Ser	–	21
	Ser/Leu	–	39
	Leu/Leu	3	14
p.Ala612Glu	Ala/Ala	–	12
	Ala/Glu	–	44
	Glu/Glu	3	18
*LYST* p.His2015Arg	His/His	3	67
	His/Arg	–	5
	Arg/Arg	–	2
p.Ala2575Val	Ala/Ala	1	44
	Ala/Val	2	11
	Val/Val	–	19

All the diluted cattle possessed same genotypes of the polymorphisms in *PMEL* gene. While parts of standard cattle had the same genotypes of p.Gly22Arg, p.Ser36Leu, and p.Ala612Glu with the diluted cattle, the del/del type of p.Leu18del was detected only in the diluted cattle. This data strongly suggested *PMEL* p.Leu18del was the causative of coat color dilution in Kumamoto sub-breed of Japanese Brown cattle (Table 2).

### Coat color dilution and PMEL p.Leu18del

**Fig. 1:**
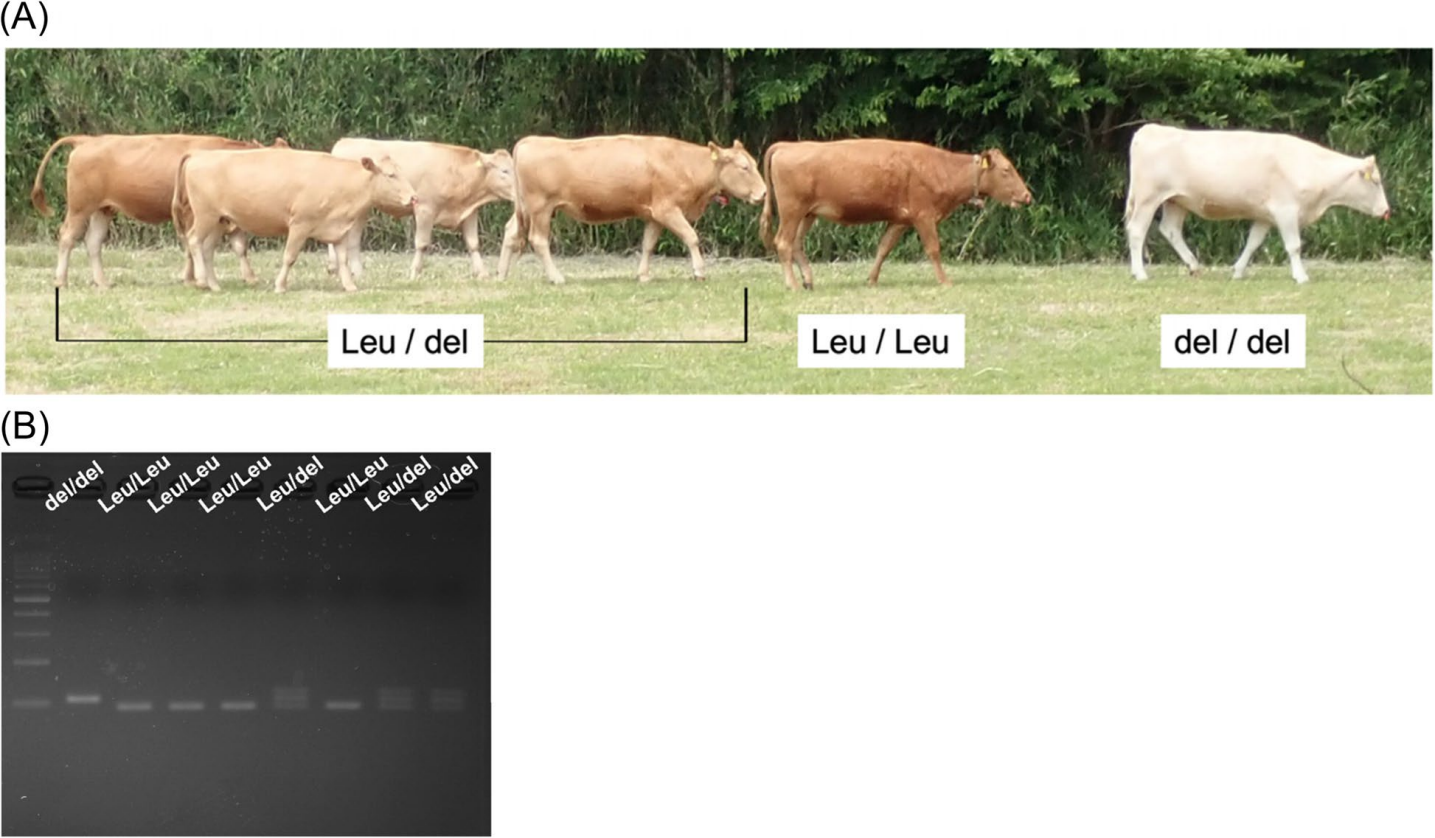
Coat color of Kumamoto sub-breed of Japanese Brown cattle and *PMEL* p.Leu18del. **A** Standard coat color of Kumamoto sub-breed is solid brown, although individuals with abnormal, diluted coat color appear occasionally. **B***PMEL* p.Leu18del could explain this phenotype. Individuals with Leu/Leu type showed standard coat color, while del/del cattle diluted one. Coat colors of heterozygotes were intermediate with various dilution rates, suggesting this phenotype is inherited in an incomplete fashion

To analyze the effect of *PMEL* p.Leu18del on coat color, genotyping was conducted with 21 individuals of Kumamoto sub-breed, containing four diluted individuals, in Aso Farm. In this family, all the individuals with diluted coat color were del/del type (Fig. S1). Although individuals with standard coat color had Leu/Leu or Leu/del type, coat colors of heterozygotes were intermediate with various dilution rates, suggesting this phenotype is inherited in an incomplete manner (Fig. 1). Although p.Leu18del is suggested to cause hypotrichosis, hereditary hair loss, in Hereford and Holstein-Friesian crossbreeds [31], such the phenotype was not observed in the individuals with del/del type in Kumamoto sub-breed.

## Discussion

The current study identified *PMEL* p.Leu18del as the responsible mutation for coat color dilution in Kumamoto sub-breed of Japanese Brown cattle. This gene encodes a pre-melanosome protein to form amyloid fibers which function as scaffolds in melanin deposition [32]. PMEL protein is first translocated to endoplasmic reticulum and then undergoes multiple modifications to form amyloid fibrils [33]. *PMEL* p.Leu18del was identified in the signal peptide domain, essential for translocation, suggesting that amyloid fibrils formation might be disrupted in the diluted cattle, because del/del type of PMEL protein could not translocate to endoplasmic reticulum. In fact, *DSPP* (*Dentin Sialophosphoprotein*) p.Tyr6Asp in the signal peptide region abolishes the signal peptide function, and prevents DSPP protein from entering endoplasmic reticulum, resulting in dentin dysplasia [34].

Mutations in the signal peptide domain of bovine *PMEL* gene have been reported to dilute coat color in several cattle breeds. The del allele of p.Leu18del has been reported as the polymorphism causing coat color dilution in Highland and Galloway cattle [29]. Artificially del allele introduced Holstein-Friesian cattle have also shown coat color dilution [35]. p.Gly22Arg is another polymorphism identified in the signal peptide domain, which was suggested to be involved in coat color dilution of Charolais and Holstein-Friesian crossbred cattle [30]. Although the Arg allele has been reported to dilute coat color, our results indicated that this polymorphism was not involved in the diluted phenotype of Kumamoto sub-breed, because individuals with standard coat color possessed Arg/Arg type.

In mice, *PMEL* gene is responsible for deposition of brown or black eumelanin, not yellow or red pheomelanin [22]. Brown, standard coat color of Kumamoto sub-breed is derived from the genotype of *MC1R* (*Melanocortin 1 receptor*) gene, which encodes a receptor for α-melanocyte-stimulating hormone to determine which pigment is produced. Individuals in this sub-breed possess *e* allele (deficient type) of *MC1R* c.310G > - and/or A allele of c.871G > A, both of which are suggested to be loss-of-function mutations to predominantly produce pheomelanin [36]. Although *PMEL* gene does not regulate pheomelanin synthesis [22], our data showed that the *PMEL* abnormality might cause coat color dilution of this sub-breed in a dose-dependent manner. That’s maybe because *MC1R* deficiency does not stop eumelanin synthesis completely; coat of cattle with *e*/*e* type of c.310G > - contains a small amount of eumelanin [37]. Therefore, decrease of eumelanin ratio caused by the *PMEL* polymorphism might dilute coat color of Kumamoto sub-breed.

Kumamoto sub-breed of Japanese Brown cattle was developed by crossing with imported cattle (mainly Simmental cattle) [38], suggesting del allele of *PMEL* p.Leu18del was derived from these imported breeds. Actually, the intensity of coat color in Fleckvieh cattle, developed from Simmental cattle, is controlled by the genome region around the *PMEL* gene [39], although the responsible polymorphism is not elucidated. On the other hand, the del allele was detected in Highland, Galloway, and cross-breed of Hereford and Holstein-Friesian cattle [29, 31]. Because Highland and Galloway cattle are Scottish origin, they might share the del allele from their ancestor. However, analysis with 19 microsatellite markers revealed that the genetic distance among Hereford, Highland, Simmental, and Holstein-Friesian cattle is not close [40]. This data suggests that the del allele of *PMEL* p.Leu18del occurred in the common ancestors of these cattle and that this allele may be present in various cattle breeds. Therefore, *PMEL* p.Leu18del can be used as a DNA marker to control cattle coat color.

## Conclusion

The del/del type of *PMEL* p.Leu18del diluted coat color of Kumamoto sub-breed of Japanese Brown cattle. Coat color of heterozygotes was intermediate with various dilution rates, suggesting this phenotype might be inherited in an incomplete manner. Because the del allele of p.Leu18del has been detected in genetically distant cattle breeds, this polymorphism can be used as a DNA marker to control cattle coat color.

## Materials and methods

### Animals

The genomic DNA samples used in this study were extracted from each bovine tissue using the standard phenol-chloroform method. One of three diluted individuals in Japanese Brown cattle, bred in Kumamoto Prefectural Agricultural Research Center and Kumamoto Prefectural Agricultural University, was chosen for whole-genome analysis. To identify the diluted cattle-specific polymorphisms, we also analyze two groups of cattle with standard coat colors, pooled samples of five Japanese Brown cattle and five Japanese Black cattle. These cattle were selected considering consanguinity. Genomic DNA samples for this experiment were derived from meat which commercially purchased from Toyozumi shokuniku, a meat store in Kumamoto Prefecture.

Subsequently, we genotyped candidate polymorphisms (*USP13* p.Met1Val, *PMEL* p.Leu18del, p.Gly22Arg, p.Ser36Leu, p.Ala612Glu, and *LYST* p.His2015Arg, p.Ala2575Val) for coat color dilution with the three diluted cattle. These polymorphisms were selected according to previous studies [18, 22, 24]. As negative controls, genotypes of 74 Japanese Brown cattle with standard coat color were analyzed, bred in Kumamoto Station of National Livestock Breeding Center. Genomic DNA samples of these 74 cattle were derived from blood. Animal handling was performed under the guideline of animal experiments in National Livestock Breeding Center [41].

In Aso Farm of Tokai University, 21 Japanese Brown cattle containing four diluted individuals were reared. These cattle were offspring of two sires. To analyze the effect of the most promising candidate polymorphism on coat color, genotyping with these cattle was performed. Blood samples of these cattle were collected for DNA extraction with the approval (#191051) from Institutional Animal Care and Use Committee at Tokai University. Sperm samples of the two sires were provided by Kumamoto Prefectural Agricultural Research Center. Experimental design was summarized in Table S2.

#### Whole genome sequencing

The TruSeq DNA PCR-Free kit (Illumina, San Diego, CA) was used to prepare the libraries, and whole genome sequencing was performed using Novaseq6000 (Illumina) by 150 bp paired-end reads according to the manufacturer’s workflow. Sequencing data was converted into raw data for the analysis. The Illumina sequencer generated raw images utilizing sequencing control software for system control and base calling through an integrated primary analysis software called Real Time Analysis. The base calls binary was converted into FASTQ utilizing illumina package bcl2fastq. The FASTQ was trimmed, mapped, and deduplicated by Trimmomatic-0.38 [42], bwa Version: 0.7.12-r1039 and deduplicated by picard-tools-1.48/MarkDuplicates.jar, respectively. IndelRealigner, BaseRecalibrator, HaplotypeCaller, and VariantFiltration by GenomeAnalysis TK-3.5 [43] were used for realignment, recalibration, mutation call, and mutation filtering. Annovar version.2.30 [44] was used to add mutation annotation. The realignment referred to ARS-UCD1.2 (GCA_002263795.2) as a reference sequence of bovine genome.

#### Genotyping

Genotyping for the polymorphisms identified in *USP13, PMEL,* and *LYST* genes was performed by PCR-RFLP method. *PMEL* p.Gly22Arg and *LYST* His2015Arg were genotyped by the methods that other groups developed [27, 30]. The primer sets to amplify the regions including the *USP13* p.Met1Val, *PMEL* p.Leu18del, p.Ser36Leu, p.Ala612Glu and *LYST* p.Ala2575Val were designed based on the reference sequences (GenBank NC_037328.1, NC_037332.1, and NC_037355.1, respectively) by Oligo7 (Molecular Biology Insights, Vondelpark, CO). To genotype the polymorphisms in *PMEL* and *LYST* genes, Go-Taq® (Promega Corporation, Madison, WI) was used as the PCR enzyme and PCR was performed with the following conditions: 35 cycles at 95 °C for 30 sec, annealing temperature for 30 sec, and 72 °C for 30 sec. For *USP13* genotyping, Q5 High-Fidelity DNA polymerase (New England BioLabs, Ipswich, MA) was used. To amplify this region, nested-PCR method was applied. The 1st and 2nd PCRs were performed under the following conditions: 35 cycles at 98 °C for 30 sec, annealing temperature for 10 sec, 72 °C for 30 sec. Subsequent restriction enzyme reactions were performed at 37 °C for 1 h. All restriction enzymes were purchased from New England Biolabs. Detailed information of genotyping was listed in Table 3.

**Table 3:** Primer sequences and restriction enzymes for genotyping

Polymorphism	Sequence (5′ → 3′)	T_m_ (°C)^b^	Enzyme	Size (bp)
*PMEL* p.Leu18del	F: GGAAGGAAGAACAGGATGGATCT R: TAGGGAGAGAAAAACCAGAGCAG	55	*Mbo*II	Leu: 108, 40, 18 del: 146, 18
p.Gly22Arg	F: ACTGTCAATGAGTAGCAGGATGTC R: TGCACCCAAATCTTCATGTG	60	*Sfc*I	Gly: 244, 190 Arg: 434
p.Ser36Leu	F: AAGCCACAACTACCTGAC R: TAGGCCCATCATTGCTGAC	60	*Af1*II	Ser: 492 Leu: 266, 226
p.Ala612Glu	F: AGCCAGGATCAAGACCAAG R: GATAGCTGTTAAGTAAGTGG	60	*Blp*I	Ala: 319, 144 Glu: 463
*LYST* p.His2015Arg	F: GAAAATTACAGCAGAAGTCCTTGG R: TGACAAACATAAGTATTAGTAGGAGG	60	*Fok*I	His: 66, 42 Arg: 108
p.Ala2575Val	F: TACAGATTCACTTCAGTCGCCTGCT^a^ R: TCTTGGAGGAAAATCTTCTCAAACACTAT	60	*Fun*4HI	Ala: 184, 24 Val: 208
*USP13* p.Met1Val	F-1st: TCGCCATTGGATTAAAAATAGCAGCGT R-1st TACCGGGGAGTCGTAGGAGAAGG F-2nd: CAGCGTCCCCTTCACCAG R-2nd ACTCGTTCTTGTAGACCCTG	1st: 65 2nd: 60	*Eag*I	Met: 268, 65 Val: 149, 132, 65

F, R-1st and F, R-2nd: 1st and 2nd primer set for nested PCR

^a^Underline indicates a mismatch nucleotide to introduce *Fun4H*I recognition site into the PCR product

^b^Annealing temperature for PCR reaction

## Supplementary Information

**Additional file 1: Table S1.** DNA polymorphisms in coat color-related genes identified by whole genome sequencing. **Table S2.** Number of animals used in each experiment.

**Additional file 2: Fig. S1. ***PMEL* p.Leu18del is responsible for coat color dilution in Kumamoto sub-breed of Japanese Brown cattle.

## Data Availability

The datasets generated and/or analyzed during the current study are available in the DNA Data Bank of Japan (DDBJ) and European Variation Archive (EVA) repository. Their accession numbers are DRR397986-DRR397988 and PRJEB52445, respectively.

## Abbreviations

TYR: Tyrosinase
Mlph: Melanophilin
Myo5a: Myosin VA
Rab27a: RAB27A, Member RAS oncogene family
MITF: Microphthalmia-associated transcription factor
USP13: Ubiquitin-Specific Protease 13
LYST: Lysosomal Trafficking Regulator
PMEL: Pre-melanosome protein
DSPP: Dentin Sialophosphoprotein
MC1R: Melanocortin 1 receptor
JBr: Japanese Brown cattle
JBl: Japanese Black cattle
