# Supplementary material for: PMEL p.Leu18del dilutes coat color of Kumamoto sub-breed of Japanese Brown cattle
Source: BMC Genomics. 2022 Oct 7;23:694. doi: 10.1186/s12864-022-08916-8 (PMC9541072; doi:10.1186/s12864-022-08916-8)
Supplement: Supplementary file 1 — Additional file 1: Table S1. DNA polymorphisms in coat color-related genes identified by whole genome sequencing. Table S2. Number of animals used in each experiment. [file 12864_2022_8916_MOESM1_ESM.docx]

Table S1 DNA polymorphisms in coat color-related genes identified by whole genome sequencing

| BTA | Gene | Polymorphism  (Variant ID) | Position (bp) | Ref | Alt | Diluted* | Standard^†^ | |
| --- | --- | --- | --- | --- | --- | --- | --- | --- |
|  |  |  |  |  |  |  | JBr | JBl |
| 1 | *SOX2* | p.Asn27delinsGlyAsn  - | 85303295 | T | TGGC | GlyAsn/GlyAsn | Asn/GlyAsn | Asn/GlyAsn |
|  | *USP13* | p.Met1Val  - | 87482086 | T | C | Val/Val | Met/Met | Met/Met |
| 3 | *LMX1A* | p.Ser296Pro  (rs208758396) | 3825395 | T | C | Pro/Pro | Ser/Pro | Ser/Pro |
|  | *MCOLN3* | p.Ile295Thr  (rs109745895) | 59081904 | T | C | Thr/Thr | Thr/Thr | Thr/Thr |
|  | *DOCK7* | p.Val1218Ile  (rs132869366) | 83060119 | G | A | Ile/Ile | Val/Ile | Val/Ile |
| 4 | *GPNMB* | p.Trp138Gly  (rs209910130) | 31886644 | G | T | Gly/Gly | Trp/Gly | Gly/Gly |
| 5 | *KRT75* | p.Arg29Cys  (rs134935106) | 27456406 | C | T | Cys/Cys | Arg/Cys | Arg/Cys |
|  |  | p.Ala42Val  (rs132753634) | 27456446 | C | T | Val/Val | Ala/Val | Ala/Val |
|  |  | p.Arg519Gln  (rs133464219) | 27465972 | G | A | Gln/Gln | Arg/Gln | Arg/Gln |
|  | *ADAMTS20* | p.Leu1210Pro  (rs109627988) | 37021787 | T | C | Pro/Pro | Pro/Pro | Pro/Pro |

Table S1 (continued)

| BTA | Gene | Polymorphism  (Variant ID) | Position (bp) | Ref | Alt | Diluted* | Standard^†^ | |
| --- | --- | --- | --- | --- | --- | --- | --- | --- |
|  |  |  |  |  |  |  | JBr | JBl |
| 5 | *PMEL* | p.Leu18del  (rs385468954) | 57345300 | GTTC | G | del/del | Leu/del | Leu/Leu |
|  |  | p.Ser36Leu  (rs380609136) | 57347578 | C | T | Leu/Leu | Ser/Leu | Ser/Ser |
|  |  | p.Ala612Glu  (rs378894329) | 57353147 | C | A | Glu/Glu | Ala/Glu | Ala/Ala |
|  | *MCHR1* | p.Ala336Thr  (rs110819460) | 111992145 | G | A | Thr/Thr | Thr/Thr | Thr/Thr |
| 10 | *TRPM7* | p.Ala109Val  (rs42486422) | 59677766 | T | C | Val/Val | Val/Val | Ala/Val |
| 12 | *ZIC2* | p.His419Gln  (rs381639895) | 76741966 | G | T | Gln/Gln | Gln/Gln | Gln/Gln |
|  |  | p.Val420Gly  (rs383698196) | 76741968 | G | T | Gly/Gly | Gly/Gly | Gly/Gly |
| 15 | *MPZL3* | p.Asn116Ser  (rs43573272) | 28923757 | C | T | Ser/Ser | Ser/Ser | Ser/Ser |
| 17 | *GGT1* | p.Thr234Ala  (rs41854708) | 71460641 | C | T | Ala/Ala | Ala/Ala | Ala/Ala |
| 18 | *MC1R* | p.Gly104fs  (rs110710422) | 14705684 | CG | C | fs/fs | fs/fs | Gly/Gly |

Table S1 (continued)

| BTA | Gene | Polymorphism  (Variant ID) | Position (bp) | Ref | Alt | Diluted* | Standard^†^ | |
| --- | --- | --- | --- | --- | --- | --- | --- | --- |
|  |  |  |  |  |  |  | JBr | JBl |
| 19 | *KRT17* | p.Ser66Gly  (rs43726686) | 41839863 | T | C | Gly/Gly | Gly/Gly | Gly/Gly |
| 22 | *MITF* | p.Met1Ile  (rs110881545) | 31650963 | T | C | Met/Met | Met/Ile | Met/Met |
| 24 | *SMCHD1* | p.Ser865Gly  (rs132752911) | 36988061 | A | G | Gly/Gly | Ser/Gly | Ser/Gly |
| 27 | *CASP3* | p.Tyr226His  (rs42118371) | 15050022 | A | G | His/His | Tyr/His | His/His |
|  |  | p.Val218Ala  (rs109101967) | 15050045 | A | G | Ala/Ala | Val/Ala | Ala/Ala |
| 28 | *LYST* | p.Ala2575Val  (rs208276890) | 8447847 | G | A | Val/Val | Ala/Ala | Ala/Val |
| X | *GPC3* | p.Val119Ile  (rs386088387) | 17585509 | C | T | Ile | Val/Ile | Val/Val |
|  | *NDP* | p.Ser28Asn  (rs457892968) | 99694246 | G | A | Asn | Ser/Ser | Ser/Ser |

* Male diluted individual of Japanese Brown cattle.

^†^ JBr: Japanese Brown cattle, JBl: Japanese Black cattle.

Table S2 Number of animals used in each experiment

| Procedure | Diluted | Standard* |
| --- | --- | --- |
| Whole genome sequence | 1 JBr | Pooled sample of 5 JBr, Pooled sample of 5 JBl |
| Genotyping 1^†^ | 3 JBr | 74 JBr |
| Genotyping 2 | 4 JBr | 17 JBr |

* JBr: Japanese Brown cattle, JBl: Japanese Black cattle.

^†^ The diluted cattle included the individual analyzed in whole genome sequence.
