# Supplementary material for: PMEL p.Leu18del dilutes coat color of Kumamoto sub-breed of Japanese Brown cattle
Source: BMC Genomics. 2022 Oct 7;23:694. doi: 10.1186/s12864-022-08916-8 (PMC9541072; doi:10.1186/s12864-022-08916-8)
Supplement: Supplementary file 2 — Additional file 2: Fig. S1. PMEL p.Leu18del is responsible for coat color dilution in Kumamoto sub-breed of Japanese Brown cattle. [file 12864_2022_8916_MOESM2_ESM.docx]

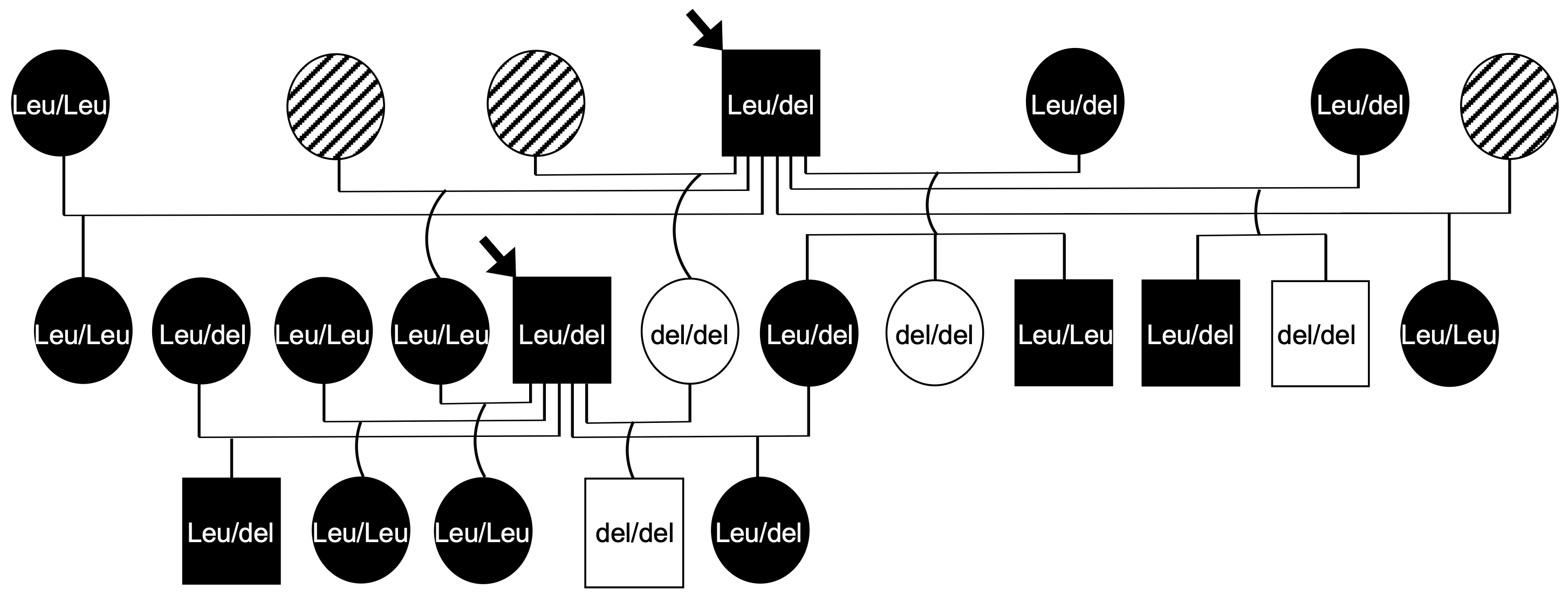


**Fig. S1 *PMEL* p.Leu18del is responsible for coat color dilution in Kumamoto sub-breed of Japanese Brown cattle.**

All individuals with diluted coat color were del/del type of *PMEL* p.Leu18del, suggesting del allele causes coat color dilution in Kumamoto sub-breed. Square: male, circle: female, black: standard coat color, white: diluted coat color, multiple diagonal lines: unknown coat color (slaughtered before this study started), arrow: sire.
